# Cognitive behavioral markers of neurodevelopmental trajectories in rodents

**DOI:** 10.1038/s41398-021-01662-7

**Published:** 2021-10-30

**Authors:** K. H. Christopher Choy, Jiaqi K. Luo, Cassandra M. J. Wannan, Liliana Laskaris, Antonia Merritt, Warda T. Syeda, Patrick M. Sexton, Arthur Christopoulos, Christos Pantelis, Jess Nithianantharajah

**Affiliations:** 1 Drug Discovery Biology, Monash Institute of Pharmaceutical Sciences, Monash University, Melbourne, VIC Australia; 2 The Florey Institute of Neuroscience and Mental Health, Melbourne, VIC Australia; 3 Department of Florey Neuroscience, University of Melbourne, Melbourne, VIC Australia; 4 Melbourne Neuropsychiatry Centre, Department of Psychiatry, University of Melbourne, Melbourne, VIC Australia; 5 ARC Centre for Cryo-electron Microscopy of Membrane Proteins, Monash Institute of Pharmaceutical Sciences, Monash University, Melbourne, VIC Australia

**Keywords:** Learning and memory, Psychology

## Abstract

Between adolescence and adulthood, the brain critically undergoes maturation and refinement of synaptic and neural circuits that shape cognitive processing. Adolescence also represents a vulnerable period for the onset of symptoms in neurodevelopmental psychiatric disorders. Despite the wide use of rodent models to unravel neurobiological mechanisms underlying neurodevelopmental disorders, there is a surprising paucity of rigorous studies focusing on normal cognitive-developmental trajectories in such models. Here, we sought to behaviorally capture maturational changes in cognitive trajectories during adolescence and into adulthood in male and female mice using distinct behavioral paradigms. C57 BL/6J mice (4.5, 6, and 12 weeks of age) were assessed on three behavioral paradigms: drug-induced locomotor hyperactivity, prepulse inhibition, and a novel validated version of a visuospatial paired-associate learning touchscreen task. We show that the normal maturational trajectories of behavioral performance on these paradigms are dissociable. Responses in drug-induced locomotor hyperactivity and prepulse inhibition both displayed a ‘U-shaped’ developmental trajectory; lower during mid-adolescence relative to early adolescence and adulthood. In contrast, visuospatial learning and memory, memory retention, and response times indicative of motivational processing progressively improved with age. Our study offers a framework to investigate how insults at different developmental stages might perturb normal trajectories in cognitive development. We provide a brain maturational approach to understand resilience factors of brain plasticity in the face of adversity and to examine pharmacological and non-pharmacological interventions directed at ameliorating or rescuing perturbed trajectories in neurodevelopmental and neuropsychiatric disorders.

## Introduction

From birth to adulthood, the vertebrate brain undergoes enormous growth and maturation that supports the development of behavior and cognition. During early childhood, brain volume significantly increases with differential growth trajectories in gray and white matter, reflecting neuronal density and myelination, respectively [1]. In adolescence and early adulthood, maturation and refinement of synaptic and neural circuitry are predominant, which are important in shaping the more specialized aspects of cognitive processing that mature by adulthood [2, 3]. Clinical and preclinical research highlights that there is still much to uncover about the rates of maturation of distinct cognitive functions, including the impact of sex on these processes, the underlying neurobiological principles that enable them, and their modulation by pharmacological and other interventions.

Childhood and adolescence are critical periods for neurodevelopment, characterized by widespread changes in brain structure [4, 5] and gradual enhancement of neurocognitive performance [3, 6]. Structural brain changes during development progress in a predictable manner, with the refinement of brain networks beginning earlier in more posterior brain regions, and the development of more anterior brain regions, such as the frontal and temporal lobes, continuing into adulthood [7]. The development of more posterior brain regions early in life supports the maturation of motor and basic cognitive skills in infancy and childhood, whereas regulation of behavior and emotion, evaluation of risk and reward, and higher-order cognitive and social functions mature in adolescence-adulthood in line with the development of fronto-medial-temporal brain networks [8]. For example, simple reaction times have been suggested to reach adult levels in early adolescence, while complex executive processes requiring the integration of multiple cognitive processes continue to mature into adolescence and adulthood [3, 6, 9–11].

Differential trajectories of brain and cognitive maturation likely represent critical factors underlying heterogeneity of dysfunction across different cognitive domains in neurodevelopmental disorders. Thus, cognitive deficits in these disorders may be shaped by the nature and timing of genetic and/or environmental insults, and the timing of illness onset [12–14]. The rapid development of fronto-medial-temporal circuits during adolescence, when neurodevelopmental and neuropsychiatric disorders such as schizophrenia emerge, renders these circuits vulnerable to insult during this period. We have proposed that a brain maturational or neurodevelopmental perspective provides an important context within which to understand the neurobiological basis of these disorders [14, 15]. Thus, brain networks and functions that are continuing to mature at the time of illness onset are more likely to be severely impacted than those that have matured prior to illness onset [15, 16]. Thus, longitudinal studies that map normal developmental trajectories of brain development and function [17] are required to understand deviations for normative trajectories when mental disorders emerge [12, 18, 19].

Critical to this endeavor are preclinical models of normal maturational trajectories that allow us to examine how the exact nature and timing of genetic/environmental insults [12, 14, 15, 20] impacts the development and refinement of brain circuits that underlie cognitive processing, and the behavioral consequences of these insults [21]. While rodent models are commonly used to examine neurobiological mechanisms underlying neurodevelopmental disorders, and to test different classes of preclinical drug candidates, there is a surprising paucity of rigorous studies focusing on normal cognitive-developmental trajectories in such models. This likely reflects limitations in the availability of cognitive tasks that can be robustly applied across the short temporal window of the rodent adolescent age range [22]; in humans, early adolescence is ∼10–12 years of age, mid-late adolescence ∼14–16 years of age and early adulthood ∼20–25 years of age, whereas in mice, corresponding ages are 4.5 weeks (W) for early adolescence, 6 W for mid-adolescence and 12 W for adulthood [22, 23].

**Fig. 1:**
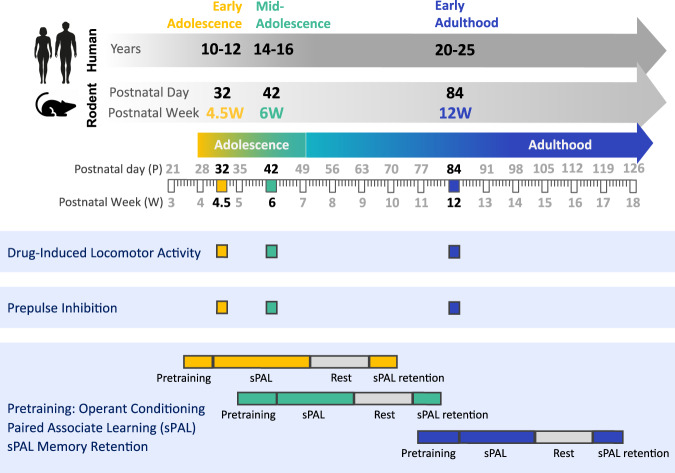
Schematic of the experimental timeline to capture behavioral trajectories during adolescence and adulthood in male and female mice on distinct cognitive tasks. In humans, early adolescence is ∼10–12 years of age, mid-late adolescence ∼14–16 years of age, and early adulthood ∼20–25 years of age. Aligning this, the behavior of mice at two adolescent ages (early 4.5 W, mid 6 W) and adulthood (12 W) was measured using drug-induced locomotor hyperactivity, prepulse inhibition, and a two-object simple paired associate learning (sPAL) touchscreen task which involved pretraining to acquire operant conditioning, sPAL training, and sPAL memory retention testing following a rest period.

Herein, we present an examination of changes in behavioral trajectories during adolescence and early adulthood in male and female mice using three distinct behavioral tasks (Fig. 1). Two of the tasks are well established behavioral assays commonly used in rodents to model symptoms of schizophrenia (e.g. [24–26]): (i) drug-induced locomotor hyperactivity is considered to reflect psychomotor agitation-like behavior observed in psychosis; and (ii) prepulse inhibition (PPI) is considered an index of sensorimotor gating and pre-attentive processing deficits described in the disorder. While commonly used in preclinical studies, the normative developmental trajectories of these tasks have not yet been examined. The third task was a relatively novel version of the paired-associate learning rodent touchscreen task [27, 28], analogous to the human paired-associate learning touchscreen task [29]. This task measures hippocampal-dependent visuospatial learning and memory; a complex cognitive process that matures during early development in humans, peaking in pre-adolescence [30]. Performance on the human version of this task is progressively disrupted in schizophrenia as the illness becomes chronic [31, 32], which parallels brain changes in the hippocampus [33].

In the present study, we show that developmental trajectories of performance on these tests are dissociable; that is, they mature normally at different rates. Drug-induced locomotor hyperactivity and PPI both displayed a ‘U-shaped’ trajectory with lower measures of performance observed during mid-adolescence compared to early adolescence and adulthood. Visuospatial learning and memory performance, on the other hand, improved with increasing age. These findings offer a framework against which to investigate how genetic and/or environmental insults at different developmental stages might perturb normal trajectories in cognitive development. They also provide an approach to the understanding of brain maturation resilience factors that respond during adversity, and to examine pharmacological and non-pharmacological interventions that may ameliorate the impact on, or rescue, trajectories that have been perturbed (e.g. [34]).

## Results

### Developmental age differentially impacts drug-induced hyperactivity and sensorimotor gating

**Fig. 2:**
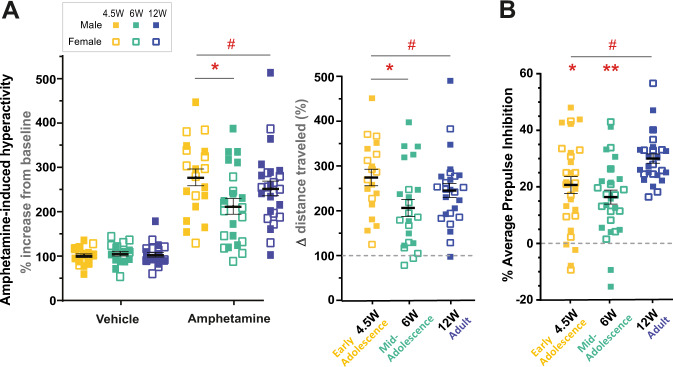
Drug-induced locomotor hyperactivity and prepulse inhibition during adolescence and adulthood. **A** Amphetamine-induced hyperactivity (% increase from pretreatment baseline (left) and % change from vehicle saline-treated animals (right)). % increase from baseline (left): Significant effect of treatment (*F*_(1,129)_ = 172.19, *P* < 0.001) and an age x treatment interaction (*F*_(2, 129)_ = 3.49, *P* = 0.034) with amphetamine inducing locomotor hyperactivity (ANOVA test with age, sex and treatment as independent factors). Examining individual treatments using a two-way ANOVA, saline vehicle-treated animals showed no effect of age (*F*_(2, 60)_ = 0.14, *P* = 0.874), sex (*F*_(1, 60)_ = 0.95, *P* = 0.334), or age × sex interaction (*F*_(2, 60)_ = 0.96, *P* = 0.391). Amphetamine-treated animals showed a significant effect of age (*F*_(2, 68)_ = 3.78, *P* = 0.028), but no sex (*F*(_1, 68_) = 1.04, *P* = 0.313) or sex × age interaction (*F*_(2, 68)_ = 2.16, *P* = 0.123). Dunnett’s multiple comparisons post hoc tests using adult mice as the reference group showed 12 W mice were not significantly different to 4.5 W (*P* = 0.514) or 6 W mice (*P* = 0.177). However, since we observed an effect of age, Bonferroni’s multiple comparisons test revealed mid-adolescent animals showed a significantly lower drug-induced locomotor response compared to early adolescent mice (*P* = 0.041). % change from vehicle saline-treated animals (right): Significant effect of age (*F*_(2,68)_ = 4.18, *P* = 0.020) but not sex (*F*_(2,68)_ = 2.42, *P* = 0.125) or age × sex interaction effects (*F*_(2,68)_ = 2.98, *P* = 0.058), two-way ANOVA. Dunnett’s multiple comparisons post hoc tests using adult mice as the reference group showed 12 W mice were not significantly different to 4.5 W (*P* = 0.391) or 6 W mice (*P* = 0.173). Since we observed an effect of age, Bonferroni’s multiple comparisons test showed mid-adolescent animals were significantly different early adolescent mice (*P* = 0.024). Data for individual mice are displayed together with mean ± SEM. #Main effect of age *P* < 0.05; *P < 0.05 between 4.5 and 6 W. Each point represents an individual mouse. **B** Percentage prepulse inhibition (average for prepulse 6, 12 and 18 combined only for the purpose of data visualization). Significant effect of age (*F*_(2,85)_ = 7.76, *P* < 0.001) but no effect of sex (*F*_(2,85)_ = 0.11, *P* = 0.746) nor age × sex interaction effects (*F*_(2,85)_ = 0.13, *P* = 0.879). Both 4.5 W (*P* = 0.018) and 6 W (*P* < 0.001) mice were significantly different to 12 W mice; repeated measures (3 prepulses) ANOVA with post hoc Dunnett’s multiple comparisons test using adult mice as the reference group. See Fig. S3B for additional analysis on effect of age on prepulse inhibition. Data for individual mice are displayed together with mean ± SEM. #Main effect of age *P* < 0.05, * *P* < 0.05, **P < 0.01 relative to 12 W sex-matched mice. Each point represents an individual mouse.

We first examined two rodent behavioral assays: drug-induced hyperactivity and PPI of acoustic startle that have been widely used to measure preclinical endophenotypes of relevance to schizophrenia symptoms. Development is accompanied by significant growth in body weight and size (Supplemental Fig. S1), which directly impacts baseline measures obtained across tests such as these (e.g. distance moved, startle amplitude). Thus, to examine the impact of age while controlling for the contribution of body weight, we focused on calculations of a relative change in performance. We first assessed baseline exploratory and locomotor activity in a novel open-field environment, then measured the hyperactivity response induced by amphetamine, which has been used as a proxy for capturing psychomotor agitation-like behavior in rodent models. When exposed to an open field, adult mice displayed significantly greater locomotor activity than early and mid-adolescent mice, with female mice moving more than male mice across all ages (Supplemental Fig. S2). Following administration of amphetamine, mice at all three ages displayed the expected amphetamine-induced locomotor hyperactivity in contrast to control mice that received the saline vehicle (Fig. 2). The change in locomotor activity following vehicle or amphetamine administration relative to baseline activity (Fig. 2A left) revealed a significant effect of treatment and an age × treatment interaction. In contrast to vehicle-treated animals that showed no differences, there was a significant effect of age following amphetamine treatment (but no sex, or sex × age interaction). Post hoc Dunnett’s multiple comparisons using the 12 W group as our reference showed that the change in drug-induced locomotor response displayed by adult mice was not significantly different from that displayed by either early or mid-adolescent mice. However, since we observed a clear main effect of age, post hoc Bonferroni’s multiple comparisons revealed mid-adolescent animals displayed significantly lower drug-induced hyperactivity compared to early adolescent mice. This same pattern was seen when analyzing the change in hyperactivity following amphetamine relative to vehicle saline treatment (Fig. 2A right).

We next examined sensorimotor gating using the prepulse inhibition test. As expected, baseline measures of acoustic startle amplitudes were different across ages (Supplemental Fig. S3A) given age-related differences in body size can impact startle measurements. As the prepulse intensity increased (i.e. PP6, 12, 18), mice at all three ages showed the expected increase in percentage inhibition (Supplemental Fig. S3B). We again observed a significant effect of age, but no differences due to sex, or any sex × age interaction. Post hoc analysis confirmed that adult mice displayed significantly greater PPI compared to both early and mid-adolescent mice (Fig. 2B).

In both the drug-induced hyperactivity and PPI tests, there were subtle suggestions for a shared pattern of performance across the three ages. Mathematical modeling of the data with age as the independent variable revealed that the data was best fit using a quadratic regression: amphetamine-induced changes in locomotor activity, *R*^2^ = 0.096, *P* = 0.035, and percentage PPI, *R*^2^ = 0.162, *P* = 0.001. This supports the observation that on both the drug-induced hyperactivity and PPI tasks, mid-adolescent mice display lower response measures than early adolescent and adult mice, and that performance on both these tests was not impacted by sex across the three age groups.

### Establishing a rapid visuospatial learning and memory test in mice to measure developmental changes

Reward-based operant tests provide opportunities to measure complex learning and memory in preclinical models. In the standard version of the rodent visuospatial learning and memory touchscreen task (‘different’ paired-associate learning, dPAL), animals learn to associate three different stimuli with each of the three touchscreen locations to form an object–location association. Training on this, and most other operant-based tasks generally requires extensive training sessions for acquisition, which can be a challenge when attempting to measure behavior within the short temporal window of adolescence in rodents.

**Fig. 3:**
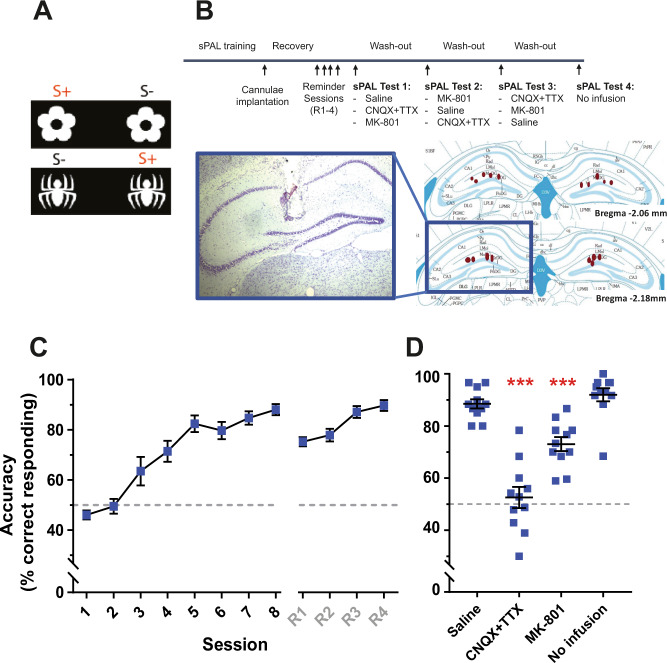
Validating hippocampal dependency for performance in the two-object sPAL touchscreen task. **A** sPAL visual stimuli (S+ rewarded, S− unrewarded). **B** Experimental sequence of hippocampal infusion study. Images show representative histological verification of cannula placement (left) and indicative sites of cannula tip placements across the group of mice tested (right). **C** Adult (12 W) male mice have trained on two-object sPAL over 8 testing sessions and showed rapid acquisition to 80% response accuracy. Animals subsequently underwent surgery to have bilateral infusion cannulae implanted into the dorsal hippocampus. Mice were given four reminder sessions (R1–R4) to reestablish pre-surgery performance levels. Data represent mean ± SEM, *n* = 11 male mice. Gray dotted line indicates performance at the chance (50% accuracy). **D** sPAL performance following dorsal hippocampal infusions with either (i) saline alone, (ii) MK-801 (NMDA receptor antagonist), or (iii) CNQX (AMPA/kainate receptor antagonist) + TTX (selective sodium channel blocker) or no infusion. Significant main effect of treatment, repeated measures ANOVA (*F*_(3, 30)_ = 48.51, *P* < 0.001). Post hoc Dunnett’s multiple comparisons test showed that compared to saline treatment, both CNQX (3 mM) + TTX (20 µM) (*P* < 0.001) or MK-801 (10 mM) (*P* < 0.001) significantly decreased performance accuracy. Performance following no infusion was not significantly different to saline treatment (*P* = 0.383). Data for individual mice are displayed together with mean ± SEM. ***P < 0.001 relative to saline. Gray dotted line indicates performance at the chance (50% accuracy).

We, therefore, assessed whether a simpler, two-object version of the touchscreen ‘same’ paired-associate learning (sPAL) task could be validated as a tool to measure the developmental trajectory of visuospatial learning during adolescence. The sPAL version only involves two identical stimuli (objects) being presented in two locations—one in the correct location and the other in an incorrect location so that animals learn a rule such that “if A, select the stimulus located on the left; if B, select the stimulus located on the right” (Fig. 3A). Thus, it is more rapidly acquired, and compatible with an assessment of adolescent development.

We first trained adult male mice on this two-object sPAL and showed that they could reach ≥80% response accuracy by the 5th training session (Fig. 3C). Previous findings had suggested that sPAL is hippocampal-dependent in male mice [28]. Validating this finding was central for our initial study, therefore due to constraints, we limited this analysis to assess males only. Following sPAL training, animals then underwent surgery to have bilateral cannulae implanted into the dorsal hippocampus. Following recovery and test sessions to reestablish stable performance (‘reminder’ sessions R1–R4, Fig. 3B), we made infusions into the dorsal hippocampus with either (i) saline, (ii) MK-801 (an NMDA receptor antagonist), or (iii) CNQX (an AMPA/kainate receptor antagonist) + TTX (a selective sodium channel blocker), and compared this to no infusion, and measured sPAL performance. We observed a significant effect of treatment, with post hoc testing confirming that relative to saline treatment, both CNQX + TTX and MK-801 significantly decreased response accuracy (Fig. 3D). Importantly, animals receiving no infusion on the last test session showed the same level of performance as saline-treated mice (Fig. 3D), confirming temporal inactivation of the hippocampus directly impacts sPAL response accuracy. These findings indicate that the hippocampus is required for visuospatial learning and memory performance on this two-object sPAL task.

### Capturing the developmental trajectory of visuospatial learning and memory in the rodent touchscreen two-object sPAL test

**Fig. 4:**
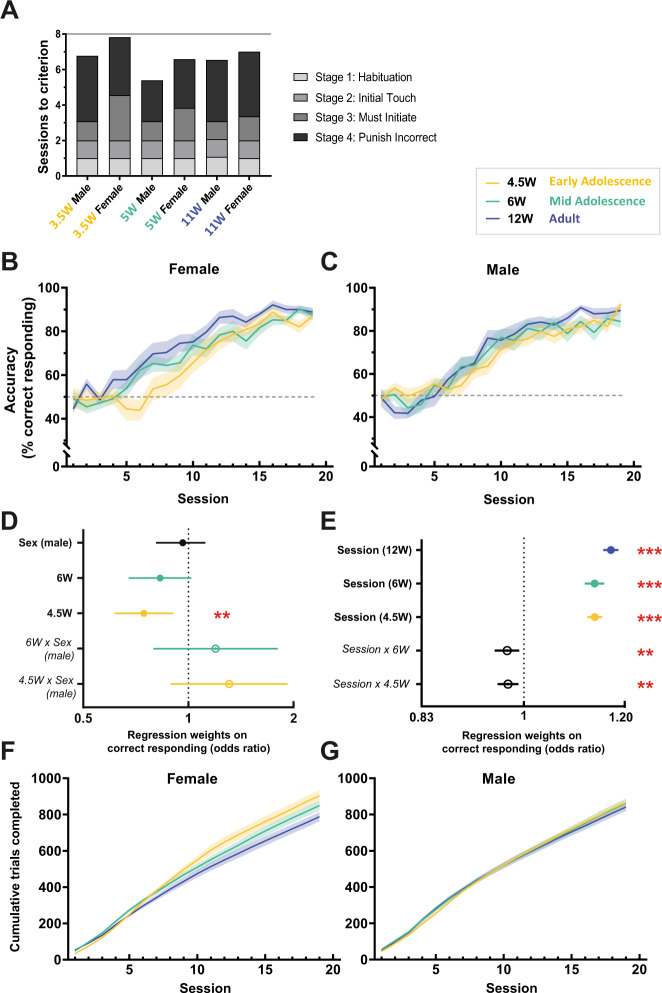
Developmental trajectory of visuospatial learning on the rodent touchscreen two-object sPAL test. **A** Touchscreen pretraining for instrumental conditioning. Mice were allocated a maximum of 8 days for pretraining and progressed through Stages 1 and 2 within comparable numbers of sessions to criterion. Female mice at all three ages, but particularly early-adolescence (3.5 W), required more training on Stage 3 compared to age-matched male mice. Data are average number of sessions at each stage (see also Supplemental Table S1). 4.5 W: *n* = 13 male, *n* = 11 female; 6 W: *n* = 13 male, *n* = 12 female; 12 W: *n* = 13 male, n = 12 female. **B** and **C** Visuospatial learning and memory in **B** female and **C** male mice on the two-object sPAL task, illustrating response accuracy (% correct) across 19 sessions. Data represent mean ± SEM. Gray dotted line indicates performance at the chance (50% accuracy). **D** and **E** Mixed-effects logistic regression analysis; Point estimates are shown with 95% CI (see Supplemental Table S2 for complete statistics). Regression weights of key biological (age, sex) and task (e.g. session) variables (denoted in filled circles), and their interaction effects (denoted in italics text, open circles) on correct responding were estimated and expressed as odds ratios. An odds ratio >1 indicates an increased likelihood of correct responding, and <1 indicates a decreased likelihood of correct responding. For regression analyses, 12 W adult mice were used as the reference age thus the effect of age (e.g. 6, 4.5 W) reflects the performance of 6 or 4.5 W mice relative to adult mice. Similarly, female mice were used as the reference sex, thus the effect of sex (e.g. male) reflects the performance of male mice relative to female mice (4.5 W: *n* = 13 male, *n* = 11 female; 6 W: *n* = 13 male, *n* = 12 female; 12 W: *n* = 13 male, *n* = 12 female). **D** There were no significant differences due to sex, nor any sex × age interactions on sPAL response accuracy. Relative to 12 W adult mice, both 6 and 4.5 W adolescent mice showed a tendency for decreased accuracy, but only 4.5 W mice were significantly different. At both 6 and 4.5 W adolescent ages, male mice tended to show better accuracy than female mice (*6* *W* *×* *Sex (male)*, *4.5* *W* *×* *Sex (male)* interaction effects), but this was not statistically significant. **P < 0.01. **E** The effect of session was used as a proxy for the rate of task acquisition to examine sPAL learning trajectories. Mice at all three ages showed changes in their learning rate across sessions, indicating acquisition of sPAL (Session (12 W), Session (6 W), Session 4.5 W)). However, relative to the 12 W adult learning trajectory, both adolescent ages showed slower rates of improvements in response accuracy (*Session* *×* *6* *W*, *Session* *×* *4.5* *W* interaction effects). **P < 0.01, *** *P* < 0.001. **F–G** Cumulative number of trials (pseudorandom first-presentation trials and correction trials) completed across sessions by **F** female and **G** male mice. Data represent mean ± SEM. Male and female data visualized separated only for clarity. Two-way ANOVA on total trials completed showed no differences due to sex (*F*_(1, 67)_ = 0.18, *P* = 0.670), or any sex × age interaction (*F*_(2, 67)_ = 1.78, *P* = 0.177), but a significant main effect of age (*F*_(2, 67)_ = 3.97, *P* = 0.024), with Bonferroni’s multiple comparisons indicating 4.5 W mice completed significantly more trials relative to 12 W animals (P = 0.020).

Using our validated two-object sPAL task, we next sought to examine the developmental trajectory of visuospatial learning and memory in male and female mice at the three developmental ages (4.5 W: early adolescence, 6 W: mid-adolescence, and 12 W: adult). Prior to learning sPAL, mice were pretrained across several stages in the touchscreen apparatus to acquire simple instrumental operant conditioning, that is, to nose-poke visual stimuli to receive rewards. To refine the length of pretraining, we simplified our previously published protocol [35–37] to only contain 4 pretraining stages to be completed within a maximum time frame of 8 days (see the section “Materials and methods”). Both male and female mice that commenced touchscreen pretraining for instrumental conditioning at 3.5, 5, and 11 W of age progressed through the first two stages within comparable numbers of training sessions. However, we qualitatively noted sex differences when animals reached the third stage, which required mice to self-initiate the commencement of trials and make an instrumental nose-poke response to stimuli in order to receive rewards (“Must Initiate”). Female mice at all three ages, but particularly early adolescence, required more sessions of training on this phase compared to age-matched male mice (Fig. 4A, Supplemental Table S1).

Following instrumental conditioning, male and female mice (now 4.5, 6, and 12 W) were trained on the two-object sPAL task to assess visuospatial learning for the same number of sessions, sufficient to allow animals in all age groups to achieve a robust level of performance accuracy (Fig. 4B, C). We analyzed response accuracy (% correct responding) across sessions using random-effect logistic regression models. For our regression analyses, 12 W adult mice were used as the reference age and female mice used as the reference sex; thus, the effect of age should be interpreted relative to adult mice and the effect of sex interpreted relative to female mice (see Methods and complete statistics provided in Supplemental Table S2). Firstly, when analyzing response accuracy, there were no significant differences due to sex, nor any sex × age interactions (see Supplemental Table S2). Secondly, performance accuracy increased with age in a graded manner, in that adult mice displayed the fastest learning, and early adolescent mice showed the slowest learning (Fig. 4B–D). Compared to adult mice, both early and mid-adolescent mice displayed poorer response accuracy (6 and 4.5 W effect size <1, Fig. 4D), which was significantly different in early adolescent mice (*P* = 0.003, Supplemental Table S2), but did not reach statistical significance in mid-adolescent mice (*P* = 0.077, Supplemental Table S2). Notably, there were no statistically significant interactions between sex and age at either adolescent age (Supplemental Table S2), highlighting no substantial effects of sex on response accuracy in visuospatial learning and memory. However, there was a tendency for male mice to perform better than female mice at both early and mid-adolescent ages (6 W × Sex (male) and 4.5 W × Sex (male) interaction effect size >1, Fig. 4D).

**Fig. 5:**
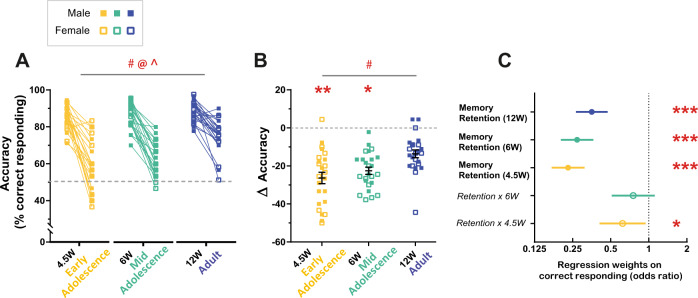
Visuospatial memory retention on the rodent touchscreen two-object sPAL test. Following sPAL training (sessions 1–19) by when mice were performing at 85–90% accuracy (Fig. 3B, C), mice were rested for 2 weeks then memory retention assessed (session 20). **A** Accuracy (% correct responding) on sessions 17–19 compared to session 20 showed significant effects of age (*F*_(2,67)_ = 13.84, *P* < 0.001), rest period on memory retention (i.e. performance at session 17–19 vs. session 20) (*F*_(1,67)_ = 243.15, *P* < 0.001) and an age × rest period interaction (*F*_(2,67)_ = 7.39, *P* = 0.001) but no significant effect of sex (*F*_(1,67)_ = 0.05_,_ *P* = 0.834). Repeated measures ANOVA. #Main effect of age *P* < 0.05, @Main effect of time on retention *P* < 0.05, ^Age × time interaction *P* < 0.05. Paired data for individual mice are displayed for mean percent correct responding prior to resting (sessions 17–19) and post 2 weeks rest (session 20). 4.5 W: *n* = 13 male, *n* = 11 female; 6 W: *n* = 13 male, *n* = 12 female; 12 W: *n* = 13 male, *n* = 12 female. Gray dotted line indicates performance at chance (50% accuracy). **B** Level of memory decay (change (Δ) in accuracy from sessions 17–19 to session 20) revealed a significant effect of age (*F*_(2,72)_ = 7.39, *P* = 0.001) but no sex effect (*F*_(2,72)_ = 1.08, *P* = 0.303) or age × sex interaction (*F*_(2,72)_ = 2.09, *P* = 0.132) (two-way ANOVA with post hoc Dunnett’s multiple comparisons). # = main effect of age *P* < 0.05, *P < 0.05, **P < 0.01 relative to 12 W sex-matched mice. Relative to adults, memory retention was decreased in 4.5 W (*P* = 0.001) and 6 W adolescent mice (*P* = 0.015). 4.5 W: *n* = 13 male, *n* = 11 female; 6 W: *n* = 13 male, *n* = 12 female; 12 W: *n* = 13 male, *n* = 12 female. Gray dotted line indicates no change in memory retention (Δ accuracy from sessions 17–19 to session 20). **C** Logistic regression analysis of the data (point estimates with 95% CI; see Supplemental Table S2 for complete statistics). Regression weights of key variables (denoted in filled circles) and their interaction effects (denoted in italics text, open circles) on correct responding were estimated and expressed as odds ratios. An odds ratio >1 indicates an increased likelihood of correct responding, and <1 indicates a decreased likelihood of correct responding. Memory retention (i.e. performance at session 20 relative to sessions 17–19) was significantly affected in all threee age groups (Memory retention (12 W), Memory retention (6 W), Memory retention (4.5 W)) with adults showing the best memory retention and early adolescent mice displaying the most impaired memory retention. This was supported by interaction effects showing that relative to 12 W adult mice (reference group), although memory retention of 6 W mid-adolescent mice was decreased (*Retention* *×* *6* *W*), memory retention in 4.5 W early adolescent mice was significantly impaired (*Retention* *×* *4.5* *W*). *P < 0.05, **P < 0.01, ***P < 0.001 (4.5 W: *n* = 13 male, *n* = 11 female; 6 W: *n* = 13 male, *n* = 12 female; 12 W: *n* = 13 male, *n* = 12 female).

To further investigate learning trajectories, we used the effect of the session as a proxy for the rate of task acquisition (Fig. 4E) and found that for all sessions, the effect of the session was significant at all our three age groups, indicating that all mice displayed sPAL learning. However, age significantly impacted the rate of acquisition and improvement in response accuracy (session × age group interactions: Session × 6 W, *P* = 0.007, Session × 4.5 W, *P* = 0.003, Supplemental Table S2). Both early and mid-adolescent mice showed slower rates of sPAL acquisition compared to adults (Fig. 4E). Separating this analysis into two blocks, sessions 1–10 (when there was no overlap in the ages of adolescent groups at the time of testing) and sessions 11–19 showed a similar pattern, supporting that early adolescent mouse displayed significantly slower rates of improvement in sPAL learning within the earlier session block (Supplemental Fig. S4). Importantly, this age effect on slower learning was not simply due to a decrease in the cumulative number of trials adolescent mice completed during testing (Fig. 4F, G). There were no significant differences due to sex, or any sex × age interaction; however, we did observe a significant effect of age with 4.5 W mice, particularly females, completing more trials relative to adult mice. These data collectively indicate that adolescent mice acquire paired-associate learning much slower than adult mice, and that performance is more negatively affected in the younger the age and in females over males, suggesting both age and sex influence the maturational trajectory of visuospatial learning. Beyond the acquisition of visuospatial learning, we were also interested in how age may impact the ability to retain the visuospatial memory following a period of time. Therefore, following two-object sPAL training, all mice were rested for 2 weeks from any experimental training then tested for memory retention of the sPAL task. The 4.5 W group were now P65 ∼9.5 W (∼20 human years), the 6 W group now P76 ∼11 W (∼22 human years), and the 12 W group now P118 ∼17 W (∼28 human years) on their first test session for memory retention. As expected, all mice initially displayed poorer performance (decreased response accuracy) on the first test session after the rest period (session 20) compared to their performance prior to the rest period (session 17–19 average) (Fig. 5A). However, the retention accuracy was lower the younger the age of the animals, (4.5 W < 6 W < 12 W) (Fig. 5A–C), with adults showing the best memory retention, and early adolescent mice displaying the most impaired memory retention. We continued to test animals for a total of 7 sessions following the rest period (sessions 20–27) and observed that with this extended training, mice from all three age groups were once again able to reach ∼90% response accuracy with no differences between ages (Supplemental Fig. S5). Collectively, these data reveal that age progressively affects visuospatial learning and memory retention on the rodent touchscreen two-object sPAL task, highlighting the importance of maturation on memory performance.

### Linking motivation with the trajectory of learning

**Fig. 6:**
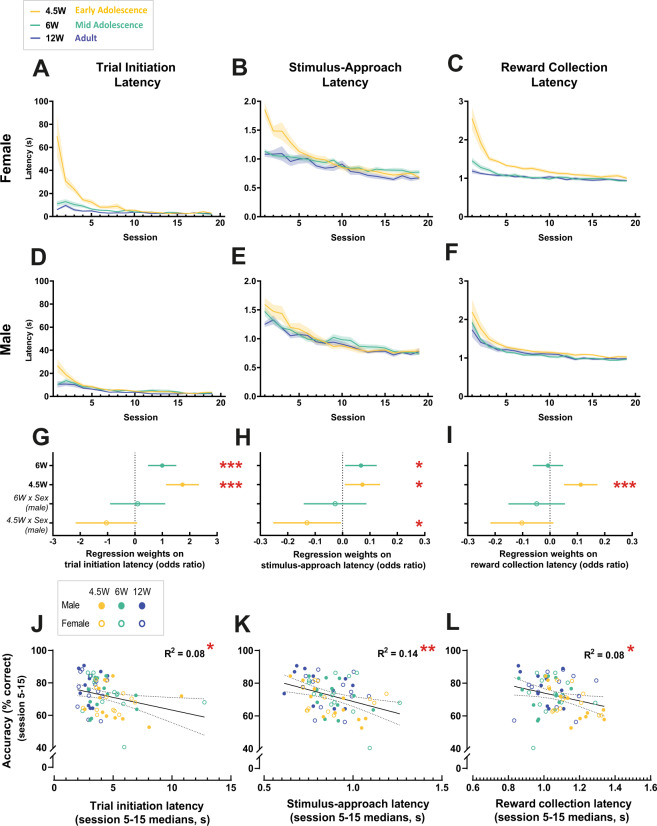
Interaction between motivation and learning across developmental ages. To measure the motivation of mice to perform the two-object sPAL, we measured latencies to initiate the commencement of a trial (trial initiation latency) (**A**, **D**, **G**), approach the touchscreen (stimulus-approach latency) (**B**, **E**, **H**), and collect rewards after a correct response (reward collection latency) (**C**, **F**, **I**) in male and female mice across the three developmental age groups. **A**–**F** Data represent mean ± SEM. **G–I** Median regression; Point estimates are shown with 95% CI (4.5 W: *n* = 13 male, *n* = 11 female; 6 W: *n* = 13 male, *n* = 12 female; 12 W: *n* = 13 male, *n* = 12 female, see Supplemental Table S2 for complete statistics). Regression weights of key variables and their interaction effects on latencies were estimated and expressed as odds ratios. An odds ratio >0 indicates an increased latency difference to perform that action, and <0 indicating a decreased latency difference. For regression analyses, 12 W adult mice were used as the reference age, thus effect of age (e.g. 6, 4.5 W) reflects the performance of 6 or 4.5 W mice relative to adult mice. Similarly, female mice were used as the reference sex, thus the effect of sex (e.g. male) reflects the performance of male mice relative to female mice. Relative to 12 W adult mice, both 6 and 4.5 W mice took significantly longer to **G** initiate trials and **H** approach the stimulus on the touchscreen, while only 4.5 W mice took longer to collect rewards (**I**). Interaction effects indicated that at 6 W, both male and female mice displayed similar latencies to initiate trials, approach the stimulus and collect rewards (*6* *W* *×* *Sex (male*)). However, at 4.5 W, male mice tended to be faster than females at all three responses (*4.5* *W* *×* *Sex (male*)) (**G**, **H**, **I**) with stimulus-approach latency being significantly different (**H**). These data indicate the tendency for early-adolescent mice in comparison to mid-adolescent and adults to have longer reaction times to perform these actions appears to be more pronounced in early adolescent female mice over early adolescent male mice. *P < 0.05, ***P < 0.001. **J–L** Linear regression between the average % accuracy pooled from mice between the steepest learning (sessions 5–15), and the average values of initiation latency, stimulus-approach latency and reward collection latency showed a significant negative correlation between the response accuracy and all three latencies. See Supplemental Table S2 for complete statistics. *P < 0.05, **P < 0.01 (4.5 W: *n* = 13 male, *n* = 11 female; 6 W: *n* = 13 male, *n* = 12 female; 12 W: *n* = 13 male, *n* = 12 female).

*Willingness* and *ability* to perform a task are intricately linked, hence we chose to assess measures of response vigor while acquiring the sPAL task across our age groups. The two-object sPAL task, like other tests within the rodent touchscreen behavioral battery, is a self-paced free operant task in which animals self-initiate to commence trials, respond, and collect rewards on their own accord. Our recent work has demonstrated that the time mice take to initiate a trial (initiation latency), approach the front of the chamber near the touchscreen prior to making a response (stimulus-approach latency), and collect rewards (reward collection latency), reliably reflect the animals’ motivation to perform various touchscreen tasks [35]. Throughout sPAL training, these three latencies revealed two consistent patterns (Fig. 6) that were not the case for another latency measure, stimulus-selection (time from arriving at the front of the touchscreen to making a selective response by nose-poking a stimulus) where we observed no differences due to age or sex (data not shown). First, relative to adult mice both early and mid-adolescent animals took significantly *longer* (varying effect sizes >0, Fig. 6G–I) to perform these three actions (with the exception of 6 W reward collection latency), with a general directional pattern where the time taken to make these responses were longer in 4.5 W > 6 W > 12 W. Second, these increased latencies in early-adolescent mice tended to be more pronounced in females (age × sex interactions effect size <0: trial initiation, 4.5 W × Sex (male) *P* = 0.068; stimulus-approach, 4.5 W × Sex (male) *P* = 0.040; reward collection, 4.5 W × Sex (male) *P* = 0.080, Fig. 6G–I). This pattern of early adolescent female mice performing worse than males is similar to the non-significant trends observed earlier with sPAL response accuracy (Fig. 4B–D) and extends the spectrum of sex differences originally noted in the acquisition of instrumental responding during touchscreen pretraining (Fig. 4A, Supplemental Table 1). Furthermore, it is important to note that while female adolescent mice tended to make *slower* responses during sPAL training relative to male adolescent mice, female mice at all three ages moved *more* and showed *higher* velocity compared to their age-matched male counterparts during baseline locomotor activity in the open-field (Supplemental Fig. S2B, C). These data suggest that the response latency changes we see during sPAL training are not simply representative of motoric capacity. To test if this relationship between the *ability* and *willingness* to perform the task holds at the individual mouse level, we examined the correlation between the average response accuracy between session 5–15, when the mice exhibited rapid learning and the average median latencies from the same sessions (Fig. 6J–L). We found modest negative correlations between response accuracy and response latencies for trial initiation, stimulus-approach and reward collection (latencies explained 8–14% of the variance in accuracy). We did not have the statistical power to investigate whether these correlations were driven by a particular subgroup of animals. Importantly, however, longer latencies in early and mid-adolescent female mice did not lead to fewer trials being completed during test sessions. In fact, they completed more trials compared to adult female mice across training sessions (Fig. 4F, Supplemental Table S2). These data suggest that the correlation between slower visuospatial learning of the two-object sPAL and reduced response vigor or motivation to perform in adolescent mice was not due to a lack of learning opportunities. Rather, that these two behavioral measures (learning rate and response times) may likely be regulated through common biological processes that mature along the same developmental trajectory.

## Discussion

In this study, we examined the normal maturational trajectories of performance on three distinct behavioral tasks in male and female mice. The tasks, which are considered relevant to cognitive models of schizophrenia, included the standard drug-induced locomotor hyperactivity and PPI tests, with the addition of a novel validated paired-associate learning rodent touchscreen task. Using these tasks, we captured differential maturational trajectories in cognitive processing during adolescence and early adulthood. Drug-induced locomotor hyperactivity and PPI both displayed a ‘U’ shaped trajectory, with lower measures of performance during mid-adolescence compared to early adolescence and adulthood and no sex differences. In comparison, visuospatial learning and memory performance in the rodent touchscreen two-object sPAL task improved progressively with age. Further, response accuracy during sPAL modestly correlated with response vigor or motivational drive. Both measures showed the same developmental trajectory, with lower accuracy and longer response times in early adolescent mice compared with adult mice.

These data show that (i) maturational trajectories in performance on these three tests are dissociable and (ii) in a test for visuospatial learning and memory, motivational drive and learning rates are lower during adolescence compared to adulthood. These findings suggest we can measure behavioral markers of neurodevelopmental trajectories using more complex cognitive tasks, such as associative memory, in mice during adolescence and into early adulthood. Our work provides new opportunities to map deviation from normal neurodevelopmental trajectories as a consequence of genetic, environmental, or chemical insults in preclinical rodent models, with the potential to examine the neurobiological substrates relevant to each behavioral domain across development. These findings could be used when assessing interventions aimed at improving cognitive dysfunction relevant to neurodevelopmental and neuropsychiatric disorders.

Drug-induced locomotor hyperactivity and PPI remain standard tests used in rodent models to measure behavioral deficits relevant to disorders such as schizophrenia (e.g. [38]). These tests have practical advantages in that they can be rapidly administered (requiring single testing sessions) and, in the case of PPI, there is analogous testing in humans (e.g. [39, 40]). Although PPI in humans can be tested as young as 8 years of age [41], there is currently no published human data that we are aware of that allows us to directly compare the trajectory of human PPI equivalent to the rodent ages investigated in this study. One study, however, measuring PPI in human subjects aged 18–88 revealed an inverted U-shaped function with age (greatest PPI at intermediate ages) [42]. In rodents, PPI has been examined at select ages, namely adolescence at mostly 6 weeks and adulthood at mostly 10 weeks of age, but these findings are spread across separate studies and models (e.g. [43–46]). Collectively, there remains a need for human and rodent studies to systematically capture behavioral measures across the lifespan from childhood, adolescence, adulthood, and ageing.

Our study is the first to examine these tasks across developmental ages in the mouse, allowing for direct comparison between age groups. Our findings for PPI are in accord with the previous individual studies in showing that sensorimotor gating develops with age, whereby adult animals display greater PPI compared to adolescent animals [43, 45, 47]. Similarly, previous work assessing drug-induced locomotor activity in rats at postnatal day 32 [48] and mice at postnatal day 35 [46] compared to adult animals (postnatal day 70) has shown similar differences to that observed between 4.5 and 12 W mice in our work. However, our findings from 6 W old mice are novel and were critical in revealing the ‘U-shaped’ developmental trajectory in this behavior.

Both locomotor activity in an open-field and PPI engage broad circuits throughout the brain. How well these tests serve to provide effective behavioral markers of specific symptoms in human disease, or as probes of specific neural circuits, is unclear. There remains a critical translational need for refinement and development of rodent assays that allow the dissection of distinct cognitive domains, and which allow homologous behavioral measures in humans from childhood to adulthood to be captured. The Cambridge Neuropsychological Test Automated Battery (CANTAB) touchscreen test for clinical assessment of visuospatial learning and memory using a Paired-Associates learning (PAL) task has been implemented from childhood to adulthood [29]. Previous work shows that visuospatial learning matures with age in humans [6], and our current work aligns with this. We have previously shown the utility of using the mouse PAL touchscreen task for enhancing translation [36, 49]. Our recent work has also highlighted that these touchscreen behavioral tools can be used to dissect reaction times and processing speed in rodents [35, 37, 50]. While learning and motivation can be dissociated as we have previously shown using our parallel assessment of learning performance and latency measures [35], in the current study we see that these two processes are correlated across the developmental trajectory in mice. This indicates that behavioral correlates of the willingness to respond and accuracy of performance can share overlapping biological and computational processes [51–54]. Of note, our findings highlight sex-dependent effects in motivational processing and learning along the developmental trajectory from adolescence to early adulthood in mice, which we did not observe for drug-induced locomotor hyperactivity or PPI. This work provides direct evidence to support the growing call for assessment of both males and females in future preclinical rodent studies [55].

We anticipate that the behavioral approaches presented herein will provide a foundation to expand our understanding of psychiatric disorders from a brain maturation or neurodevelopmental perspective. In particular, they will provide a baseline against which to compare preclinical models of neurodevelopmental disorders, including maternal immune activation and genetic modification models. Here, we sought to develop a neurodevelopmental model using tasks that have been validated in humans and rodents, which also inform our understanding of disorders during maturation, such as schizophrenia and an autism spectrum disorder. Whilst PPI and psychostimulant-induced locomotor hyperactivity are standard measures used as potential models of schizophrenia, no prior study has mapped the PAL task across development, which we have demonstrated is relevant to schizophrenia and integrity of the hippocampus and its subfields [31, 32]. Translational models of this kind provide an opportunity for cross-species examination of the impact of brain maturation on task performance, and comparison with models of proposed insults relevant to neurodevelopmental disorders. Further, these methods provide a basis to dissect the neurobiology of these disorders at multiple levels of analysis, including the involvement of genes and environment on the development and refinement of brain circuits that underlie cognitive processing, and an impetus to explicitly incorporate the stage of neurodevelopment when assessing novel preclinical interventions.

## Materials and methods

### Animals

Time mated pregnant C57BL6/J female mice (embryos E12–14) were shipped from colonies established at Monash University (Clayton, Australia) to two experimental sites (PC1 facilities): Monash Institute of Pharmaceutical Sciences, or The Florey Institute of Neuroscience and Mental Health, Melbourne Australia. Transferring pregnant females to separate experimental sites could differentially impact mothers and embryos/offspring, therefore to reduce such confounds, identical transport procedures were used with transport time to both experimental facilities being comparable. Importantly, this design ensured that pregnant C57BL6/J female mice were obtained from the same source, which was a priority. At both experimental sites, offspring were born and then weaned at postnatal day (P) 19–21, and maintained in group-housed cages of 2–5 mice per cage in reversed light–dark lighting conditions (lights off 07:00; lights on 19:00) in temperature and humidity-controlled rooms. Mice were housed in open-top cages with woodchip sawdust for bedding containing tissues for nesting. Food and water were available ad libitum with exceptions detailed below. All behavioral testing was completed in the active, dark phase of the light cycle. These conditions were the same across both experimental sites. Additionally, the same experimenter was responsible for the husbandry and care of the animals, in addition to completing all the studies at both experimental sites.

Male and female mice were allocated to three different age groups: Early adolescence (4.5 Weeks (W), P32), Mid-adolescence (6 W, P42), and Adults (12 W, P84). A total of four cohorts of animals were used for this study: Cohort 1 (male and female mice, three ages; locomotor open-field), Cohort 2 (male and female mice, three ages; PPI), Cohort 3 (adult male mice only; two-object sPAL validation), Cohort 4 (male and female mice, three ages; two object sPAL). Note, we designed our study to have large numbers of time-mated females so that for Cohorts 1, 2, and 4 described above, which required mice of three different ages, all animals within a cohort were born at the same time. Animals were then allocated pseudorandomly across the three ages so that littermates were represented as evenly as possible across the age groups.

All experiments were conducted in accordance with the Australian Code of Practice for the Care and Use of Animals for Scientific Purposes, and approved by The Florey Institute of Neuroscience and Mental Health and/or Monash Institute of Pharmaceutical Sciences Animal Ethics Committees.

### Amphetamine-induced locomotor activity in the open-field test

Testing was carried out at Monash Institute of Pharmaceutical Sciences. Cohort 1 (4.5 W (P31): *n* = 22 male, *n* = 18 female; 6 W (P42): *n* = 21 male, *n* = 21 female; 12 W (P84): *n* = 24 male, *n* = 24 female) were tested in an open-field arena (40 cm × 40 cm white square box, central zone was inner 20 cm × 20 cm; lighting intensity <10 lx) similar to that previously described [43, 46] with modifications. Mice were placed in the arena for 60 min to measure baseline activity, then pseudorandomly allocated to receive a single administration of saline (0.1 ml/10 g i.p.; 4.5 W: *n* = 11 male, *n* = 9 female; 6 W: *n* = 9 male, *n* = 10 female; 12 W: *n* = 11 male, *n* = 11 female) or amphetamine (2.5 mg/kg i.p.; 4.5 W: *n* = 11 male, *n* = 9 female; 6 W: *n* = 12 male, *n* = 11 female; 12 W: *n* = 13 male, *n* = 13 female) and locomotor activity measured for a further 90 min. Several measures can be obtained using the video tracking software (BiObserve^®^ Viewer III, GmbH, Germany): distance traveled (cm), % time in central zone, velocity (cm/s), % activity (% time active with activity defined as movements >0.1 cm/s), ambulation and total bouts of spontaneous acceleration (>0.5 cm/s).

### Prepulse inhibition

Testing was carried out at Monash Institute of Pharmaceutical Sciences. Cohort 2 (4.5 W (P31): *n* = 16 male, *n* = 13 female; 6 W (P42): *n* = 15 male, *n* = 14 female; 12 W (P84): *n* = 16 male, *n* = 12 female) were tested for PPI. We used a modified protocol from our earlier work [34] where only a single startle pulse (120 db) was employed as we previously noted both 100 and 110 db showed the same results as 120 db. PPI testing was carried out in a sound-attenuated room using the SR-LAB startle chambers (San Diego Instruments, San Diego, CA, USA) containing an upgraded control box with USB interface (running Microsoft Windows^®^10) and thin-walled super sensitive enclosures (5″ length × 1.5″ internal diameter). A habituation PPI session was given 24 h prior to the actual test to acclimatize animals and reduce potential confounds due to novelty and stress, similar to previous work using a habituation session to the startle chamber with and without exposure to the startle stimulus [56–59]. Our data confirmed we see no differences in % PPI during habituation and test sessions (see Supplemental Fig. 3C). The PPI session lasted 35 min and consisted of randomized trials of startle alone pulses (120 db), and pulses preceded by 6, 12, or 18 db prepulses (pp6, 12 and 18) above a 65 db background white noise for each pulse. Inter-trial intervals (ITI) were on average 15 s (varying between 7 and 23 s), prepulse–pulse (inter-stimulus) interval was 100 ms, length of prepulse was 20 ms and pulse was 40 ms [60]. Acoustic startle inhibition was calculated using the formula [(pulse alone−prepulse trial)/pulse alone]×100%.

### Touchscreen food restriction and pretraining

Testing was carried out at The Florey Institute of Neuroscience and Mental Health. Cohort 4 (4.5 W: *n* = 13 male, *n* = 11 female; 6 W: *n* = 13 male, *n* = 12 female; 12 W: *n* = 13 male, *n* = 12 female) started food restriction when mice were P22, P32 or P74, and 2 days later, commenced pretraining or instrumental operant conditioning in the touchscreen apparatus at P24, P34, or P76. Adult mice in Cohort 3 (*n* = 11 male) and Cohort 4 were food-restricted to ~85–90% of free-feeding weight as previously published [35–37]. However, for the adolescent groups, we documented daily body weights and amounts of food consumed by free-feeding mice from P22 to P74 (Supplemental Fig. S1) and this was used as a guide to maintain adolescent body weights at 85–90% along the developmental growth curve. It is noteworthy that both adolescent and adult mice normally consume ~3–4 g of chow per mouse per day. Mice were maintained at ~85–90% body weight for the duration of the experiment and weighed daily (7 days/week).

Pretraining and two-object sPAL training was conducted using mouse touchscreen operant chambers (Campden Instruments Ltd, UK). For all touchscreen training, mice were tested daily, 7 days per week. We refined and simplified our previously published pretraining protocol [35–37] to only contain 4 stages. In Stage 1 (Habituation, H), mice were habituated to the touchscreen chambers by being placed in chamber for 30 min for one day and required to consume 200 µl of liquid reward freely available in the reward receptacle. Strawberry milk (Devondale, Australia) was used as the liquid reward for all touchscreen testing. In Stage 2 (Initial Touch, IT) or the Pavlovian stage, a single graphic black and white visual stimulus was displayed on the screen for 30 s, after which, the disappearance of the stimulus coincided with the presentation of a tone, illumination of the reward receptacle and delivery of the liquid reward (20 µl). If mice nose-poked the stimulus before 30 s had elapsed, mice were rewarded with 3 times the reward amount to encourage responding to stimuli on the screen. The session ended after 30 trials or a maximum of 60 min has elapsed, whichever occurred first. In Stage 3 (Must Initiate, MI), mice had to make a head-entry into the reward receptacle to initiate a new trial and nose-poke a visual stimulus that appears on the touchscreen to obtain a reward. Mice were required to complete a minimum of 24/30 trials within 60 min. Lastly, Stage 4 (Punish Incorrect, PI) was designed to discourage non-selective screen responding where nose-poke responses at a blank part of the screen during stimulus presentation now produced a 5 s timeout (signaled by illumination of the house-light and no delivery of reward). If another response to a blank part of the screen during stimulus presentation was made, there was a 5 s inter-trial interval (ITI), and then the same trial was repeated (the same stimulus presented in the same screen location, termed a correction trial) until the mouse made a correct response. Mice were required to complete a minimum of 20/30 trials per session within 60 min, with ≥70% accuracy. Stages 2–4 consisted of a maximum of 30 trials (pseudorandom first-presentation), and Stage 4 included an unlimited number of correction trials. Following mice completing all phases, or a maximum of 8 pretraining days, all mice were subsequently moved on to two-object sPAL training. Note, despite not all mice completing the criterion for MI, we ensured mice at minimum received one session of PI (i.e. on the 8^th^ pretraining day). Mice that successfully completed all Phases in under 8 days were rested so that all mice could commence sPAL training on the same day.

### Two-object same Paired Associates Learning (sPAL) task

Extending previous work [28], we used the two-object sPAL task containing two visual stimuli (flower and spider) and two locations (left and right windows of a 3-window mask). sPAL training commenced from P31/32 (4.5 W), P42 (6 W) or P84 (12 W). Mice were tested for 19 daily sessions. Sessions 1–3 required completing a maximum of 20 trials within 60 min, and all remaining sessions required 30 trials within 60 min. To assess sPAL memory retention, after 19 sessions of sPAL training, mice were rested and placed back on free-feeding and monitored daily. After 12 days food-restriction resumed, and following another 2 days (total of 14 days rest), mice (4.5 W group now P65, ∼9.5 W; 6 W group now P76, ∼11 W; 12 W group now P118, ∼17 W) were tested for memory retention on two-object sPAL for 7 sessions (i.e. sessions 20–26).

### Hippocampal infusions during two-object sPAL

Cohort 3 (12 W male mice, *n* = 11) were pretrained and trained on sPAL as that described above until mice reached a stable performance of ∼90% of accuracy. Mice were placed back on free-feeding prior to stereotaxic surgery under gaseous anesthesia (5% induction, 2–3% maintaining, surgery duration 30–45 min) to bilaterally implant custom made double guide cannula (22 gauge, −1.5 mm DV; Plastic One/BioSci Pty Ltd., Australia) to the dorsal hippocampus secured by self-cure dental cement (Henry Schein^®^ Halas, NSW, Australia) and two screws (1.6 mm length) as anchors drilled into the skull. Our target area for cannula placement ranged between −1.82 and −2.30 mm AP to bregma, ±1.5 mm ML, and −2.3 mm DV between CA1 and the upper blade of dentate gyrus [61]. Mice received meloxicam at 3 mg/kg (i.p.) per day for 2–3 days for post-surgical analgesia. No aversive clinical signs or mortality was seen. Mice were allowed to recover for 7 days within which time food-restriction resumed and then mice were given 3–4 reminder sPAL sessions.

On infusion testing days, either saline, a cocktail of 3 mM CNQX and 20 µM TTX (dose based on [28, 62]), or 10 mM MK-801 (dose based on [27]) was administered to every mouse in a pseudorandom order via infusion double cannula (28 gauge, +0.8 mm i.e. 2.3 mm DV, Plastic One). Infusion rate was 0.5 µl/side over 2 min, and an additional 1.5 min was allowed for diffusion prior to the withdrawal of the infusion cannula. After 30–45 min following infusions, mice were placed in the touchscreens to commence two-object sPAL testing. Between testing days, 2–3 rest days for drug wash-out was allocated prior to all mice receiving a final no-infusion sPAL test session. Mice were transcardially perfused with 4% PFA and 16 µm brain sections were collected to validate infusion sites using cresyl violet (2% aqueous) staining. Of note, a total of *n* = 15 mice were trained and had cannulation surgeries but only *n* = 11/15 mice were confirmed to have accurate cannulae placement (Fig. 2B), thus only these *n* = 11 mice have been included in the behavioral analysis.

### Data analysis

Locomotor open-field and PPI data were analyzed by ANOVA using SPSS statistical software (ver. 26, IBM, Armonk, NY, USA), following by post hoc Dunnett’s multiple comparisons (two-sided), with 12 W animals as the reference age and sex as the independent factor. Percentage increase in amphetamine-induced hyperactivity was calculated relative to the performance of saline treated mice (from same age and sex). For PPI, the 3 prepulses (PP6, 12 and 18) were also used as a within subject factor. The effect of hippocampal infusions on two-object sPAL performance was analyzed using a repeated-measures ANOVA followed by post hoc Dunnett’s multiple comparisons tests, where % correct was the dependent variable and drug treatment the repeated variable.

For trial-level sPAL data analysis, the binary outcome of a given trial (1 = correct/0 = correct) was modeled with mixed-effects logistic regression using STATA (ver. 15, Stata Crop., TX, USA) as previously described [35, 37]. Mice were treated as level-2 clusters and random intercepts. Latency (trial initiation, stimulus-approach, reward collection) data were modeled using median regression with robust and clustered standard errors clustered by mouse [63]. The effect size of biological (age, sex) and task variables (session, trials within a session, correct stimulus etc.) were estimated together with 95% confidence intervals (CI) and statistical significance. Effect sizes from logistic regression were exponentiated and expressed as odds ratios. An odds ratio of >1 indicates a significant increase in the likelihood of correct responding, and <1 indicates a significant decrease in the likelihood of correct responding. The effect sizes from median regression indicate the estimated change in latencies, therefore odds ratio >0 indicates an increased latency difference to perform that action, and <0 indicating a decreased latency difference.

For all data analysis, *P* < 0.05 was accepted as statistically significant.

## Supplementary information

Supplemental Data
