# Supplementary material for: Cognitive behavioral markers of neurodevelopmental trajectories in rodents
Source: Transl Psychiatry. 2021 Oct 30;11:556. doi: 10.1038/s41398-021-01662-7 (PMC8557208; doi:10.1038/s41398-021-01662-7)
Supplement: Supplementary file 1 — Supplemental Data [file 41398_2021_1662_MOESM1_ESM.pdf]

SUPPLEMENTAL DATA

Cognitive behavioral markers of neurodevelopmental trajectories in rodents

KH Christopher Choy[1], Jiaqi K Luo[2,3], Cassandra MJ Wannan[4], Liliana Laskaris[4], Antonia Merritt[4], Warda T Syeda[4], Patrick M Sexton[1,5], Arthur Christopoulos[1,5*], Christos Pantelis[2,4*], Jess Nithianantharajah[2,3*]

[1]Drug Discovery Biology, Monash Institute of Pharmaceutical Sciences, Monash University, Victoria, Australia.
[2]The Florey Institute of Neuroscience and Mental Health, Melbourne, Victoria, Australia.
[3]Department of Florey Neuroscience, University of Melbourne, Melbourne, Victoria, Australia.
[4]Melbourne Neuropsychiatry Centre, Department of Psychiatry, University of Melbourne, Victoria, Australia.
[5]ARC Centre for Cryo-electron Microscopy of Membrane Proteins, Monash Institute of Pharmaceutical Sciences, Monash University, Victoria, Australia.

*Co-corresponding authors

Arthur Christopoulos (arthur.christopoulos@monash.edu)

Christos Pantelis (cpant@unimelb.edu.au)

Jess Nithianantharajah (jess.n@florey.edu.au)

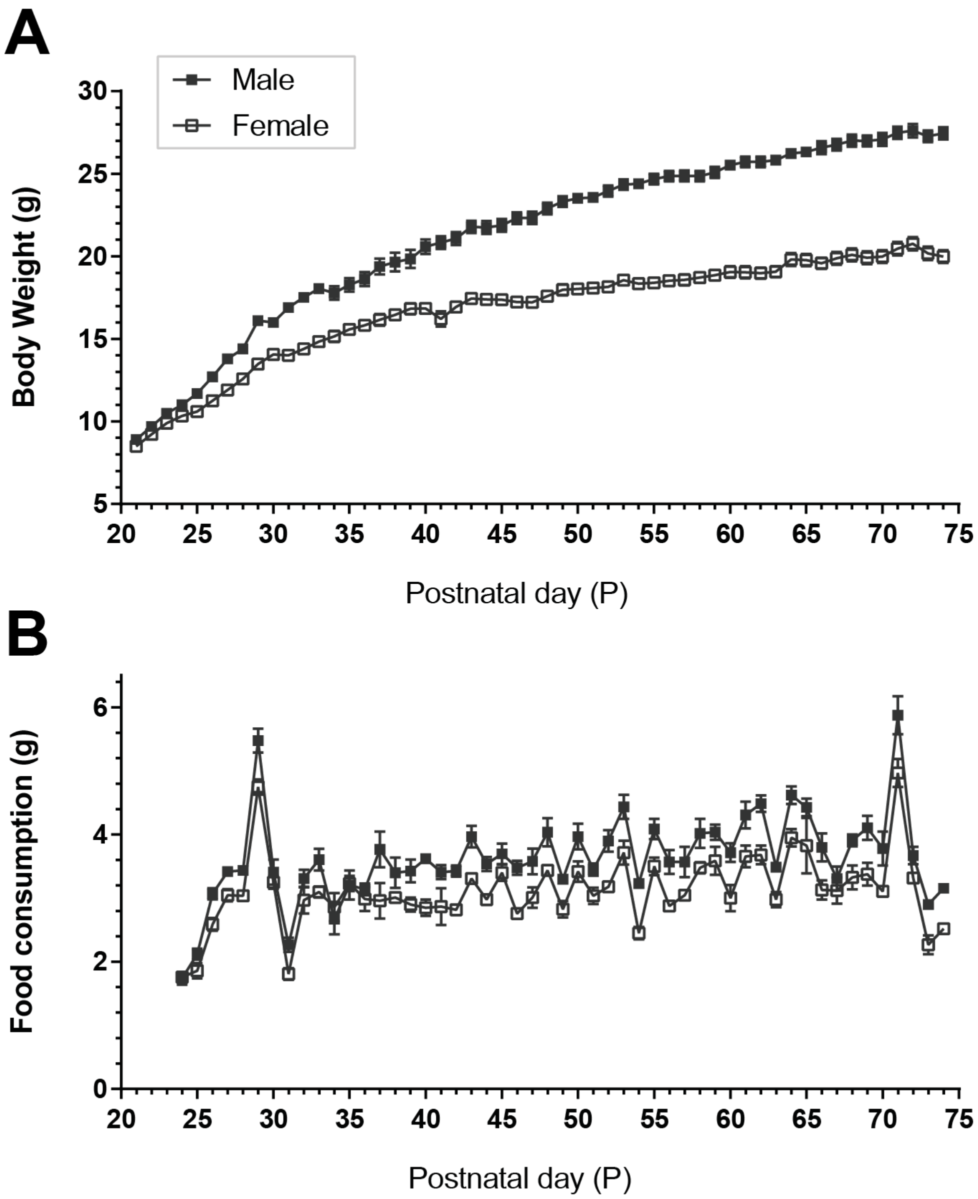

Supplemental Figure S1: Tracking natural changes body weight and food consumption (postnatal day 21-74).

Recorded daily between 08:00 and 12:00 in C57BL/6J mice with ad libitum access to food and water.

(A) Body weight in male and female mice from P21-P74.

(B) Calculation of average daily food consumption from P24-P74 (grams (g) per mouse). The home-cage lid containing the food hopper was weighed daily, and the difference from the previous day calculated and divided by the number of mice in each cage (i.e. assuming all mice group housed consumed the same quantity of food within the cage). Mice on average consumed 3-4 g of food each day and this was generally consistent from P24 to P74 (~3.5-10.5 weeks of age).

Data are mean ± SEM, n=13 male and n=12 female mice.

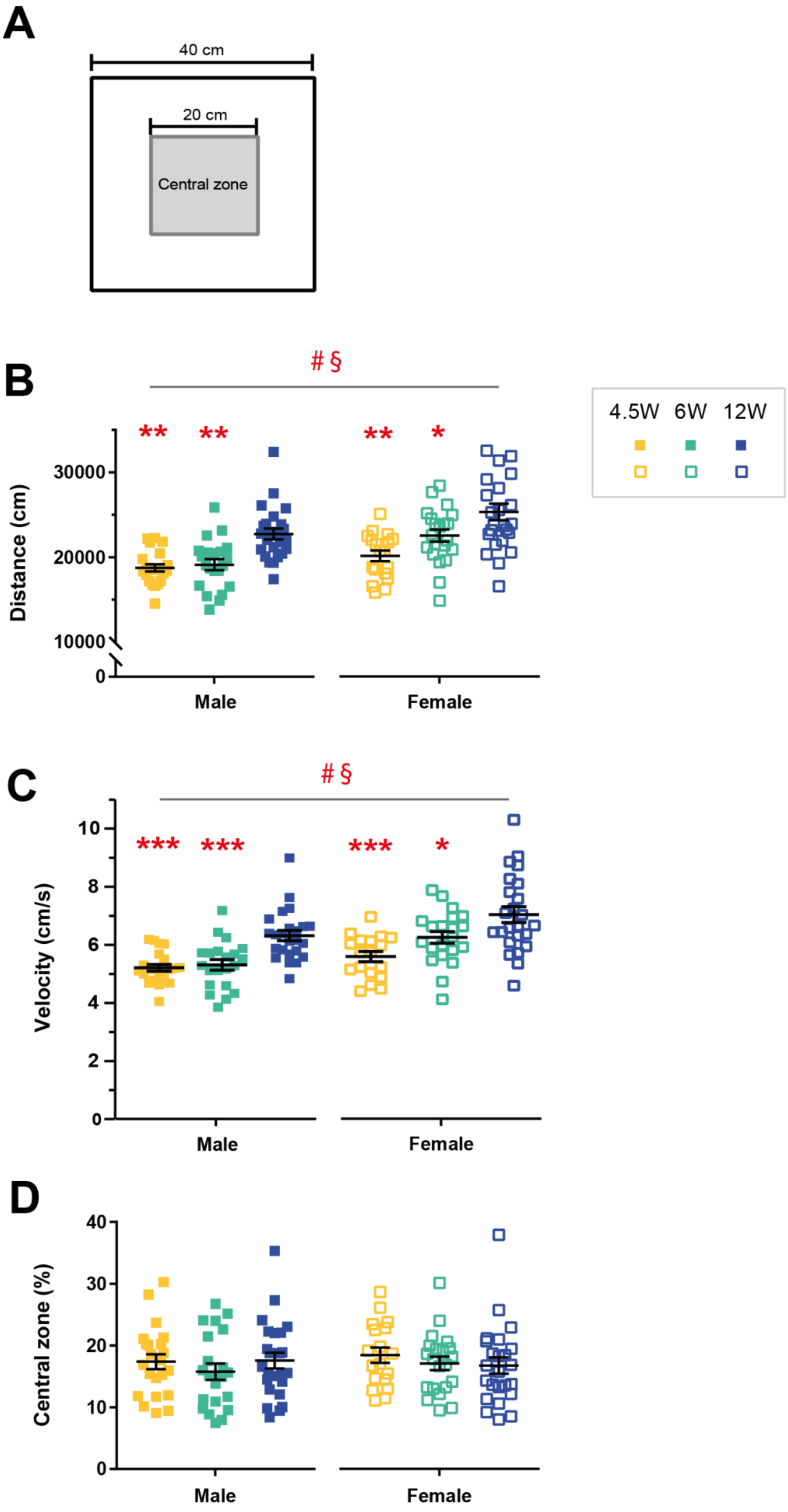
A
40 cm
20 cm
Central zone
B
§
** ** ** *
Distance (cm)
30000
20000
10000
0
Male
Female
4.5W 6W 12W
C
§
*** *** *** *
Velocity (cm/s)
10
8
6
4
2
0
Male
Female
D
Central zone (%)
40
30
20
10
0
Male
Female

Supplemental Figure S2: **Baseline exploratory activity in the locomotor open-field test.**

(A) Dimensions of the open-field arena (central and outer zones).

(B) Total distance traveled (centimeters, cm) during the first 60 minutes exposure to the open-field test. Significant effects of age ($F_{(2, 129)}$ = 22.54, $P < 0.001$) and sex ($F_{(1, 129)}$ = 17.54, $P < 0.001$) but no sex x age interaction ($F_{(2, 129)}$ = 0.95, P = 0.390). Female mice travelled greater distances compared to male mice, but both 4.5W and 6W mice traveled less distance relative to 12W adults, regardless of sex. Two-way ANOVA with post hoc Dunnett's multiple comparisons. # = main effect of age $P < 0.05$, § = main effect of sex $P < 0.05$, * $P < 0.05$, ** $P < 0.01$ relative to 12W sex-matched mice.

(C) Average velocity (centimeters, cm)/second, s) during the first 60 minutes exposure to the open-field test. Significant effects of age ($F_{(2, 129)}$ = 22.62, $P < 0.001$) and sex ($F_{(1, 129)}$ = 17.66, $P < 0.001$) but no sex x age interaction ($F_{(2, 129)}$ = 0.95, P = 0.388). Female mice showed increased velocity compared to male mice, but both 4.5W and 6W mice had decreased velocity relative to 12W adults, regardless of sex. Two-way ANOVA with post hoc Dunnett's multiple comparisons. # = main effect of age $P < 0.05$, § = main effect of sex $P < 0.05$, * $P < 0.05$, *** $P < 0.001$ relative to 12W sex-matched mice.

(D) Percentage (%) time spent in the central zone revealed no age ($F_{(2, 129)}$ = 0.65, P = 0.526) nor sex ($F_{(2, 129)}$ = 0.25, P = 0.615) dependent effects.

(B-D) Data for individual mice (4.5W: n=22 male, n=18 female; 6W: n=21 male, n=21 female; 12W n=24 male, n=24 female) are displayed together with mean ± SEM.

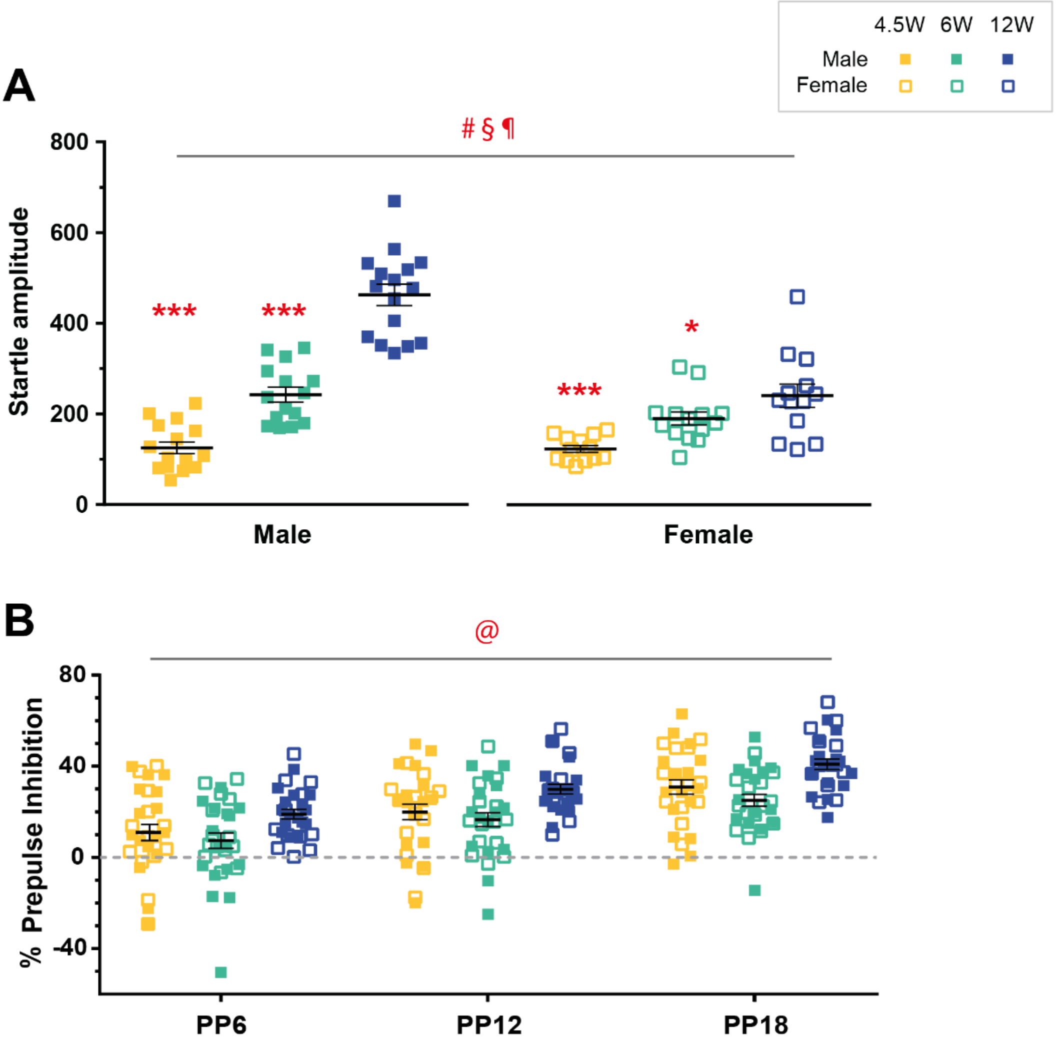

C

% Prepulse Inhibition	4.5W	6W	12W
Habituation session	22.55 ± 1.26 *	16.08 ± 2.07 ***	28.36 ± 1.72
Test session	20.66 ± 2.96 *	16.38 ± 2.49 ***	29.86 ± 1.70

Supplemental Figure S3: Startle amplitude response and prepulse inhibition.

(A) Startle amplitude showed significant effects of age ($F_{(2, 85)}$ = 83.93, $P < 0.001$) and sex ($F_{(1, 85)}$ = 36.26, $P < 0.001$), and an age x sex interaction ($F_{(2, 85)}$ = 18.38, $P < 0.001$), with startle amplitude increasing with age, and male mice showing stronger startle than female mice (ANOVA with post hoc Dunnett's multiple comparisons). Both 4.5W and 6W male mice showed decreased startle relative to 12W male mice. Similarly, both 4.5W and 6W female mice displayed significantly lower startle compared to 12W female mice. Data from individual animals are displayed together with the mean ± SEM. # = main effect of age $P < 0.05$, § = main effect of sex $P < 0.05$, ¶ = age x sex interaction $P < 0.05$, * $P < 0.05$ *** $P < 0.001$ relative to 12W sex-matched mice (4.5W: n=16 male, n=13 female; 6W: n=15 male, n=14 female; 12W: n=16 male, n=12 female).

(B) Percentage (%) prepulse inhibition at 3 different prepulses (PP6, 12 and 18) showed a significant effect of prepulse ($F_{(2, 160)}$ =75.00, $P < 0.001$) but no significant prepulse x sex ($F_{(2, 160)}$ = 0.09, $P = 0.911$), prepulse x age ($F_{(4, 160)}$ = 0.46, $P = 0.769$) or prepulse x age x sex interactions ($F_{(4, 160)}$ = 1.83, $P = 0.126$) (Repeated measures ANOVA with post hoc Dunnett's multiple comparisons, see Figure 1C for additional analyses). Data from individual animals are displayed together with the mean ± SEM. @ = main effect of prepulse $P < 0.05$.

(C) Comparison of % prepulse inhibition during habituation and test sessions. Repeated measures ANOVA (age and sex as independent factors, session (habituation and test) as repeated measures) showed no differences due to session ($F_{(1, 80)}$ = 0.01, $P = 0.941$) or session x age/sex interactions (session x age $F_{(2, 80)}$ = 0.39, $P = 0.676$; session x sex $F_{(1, 80)}$ = 0.39, $P = 0.535$; session x age x sex $F_{(2, 80)}$ = 0.19, $P = 0.829$).

PPI during Habituation: Two-way ANOVA, effect of age ($F_{(2, 85)}$ = 12.73, $P < 0.001$), effect of sex ($F_{(1, 85)}$ = 0.33, $P = 0.567$), sex x age interaction ($F_{(2, 85)}$ = 0.19, $P = 0.825$). Dunnett's multiple comparisons showed 12W vs 6W ($P < 0.001$), 12W vs 4.5W ($P = 0.036$).

PPI during Test: Two-way ANOVA, effect of age ($F_{(2, 85)}$ = 7.74, $P = 0.001$), effect of sex ($F_{(1, 85)}$ = 0.11, $P = 0.745$), sex x age interaction ($F_{(2, 85)}$ = 0.13, $P = 0.879$). Dunnett's multiple comparisons showed 12W vs 6W ($P < 0.001$), 12W vs 4.5W ($P = 0.018$).

* $P < 0.05$, *** $P < 0.001$ relative to 12W mice.

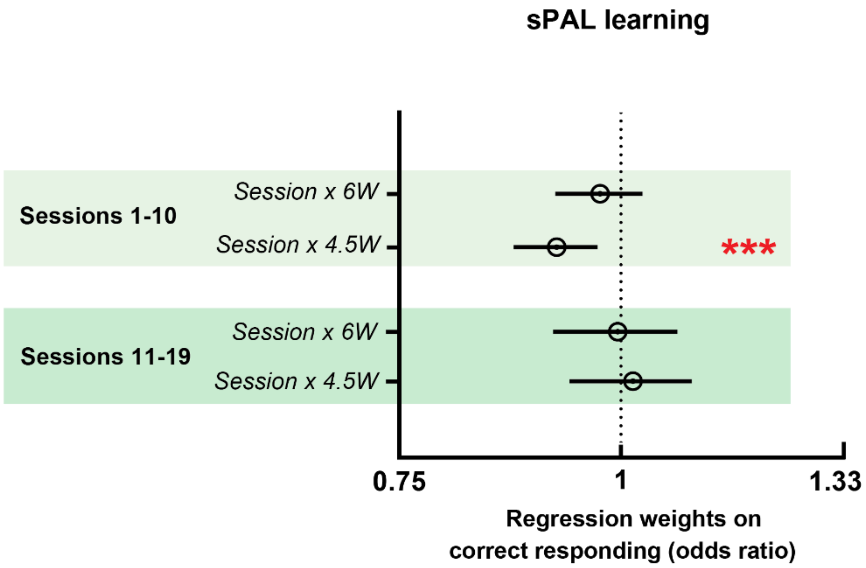

Supplemental Figure S4: sPAL learning (sessions 1-10 and 11-19)

Analyzing the effect of session (rate of sPAL learning) for sessions 1-10 (when the age of adolescent animals at the time of testing did not overlap) and sessions 11-19. During sessions 1-10, early adolescent mice showed significantly slower rates of improvement in response accuracy relative to 12W adult mice. In comparison, adolescent mice showed no differences to 12W adult mice in the rates of learning during sessions 11-19. ***$P < 0.005$. Point estimates are shown with 95% CI, see Supplemental Table S2 for complete statistics.

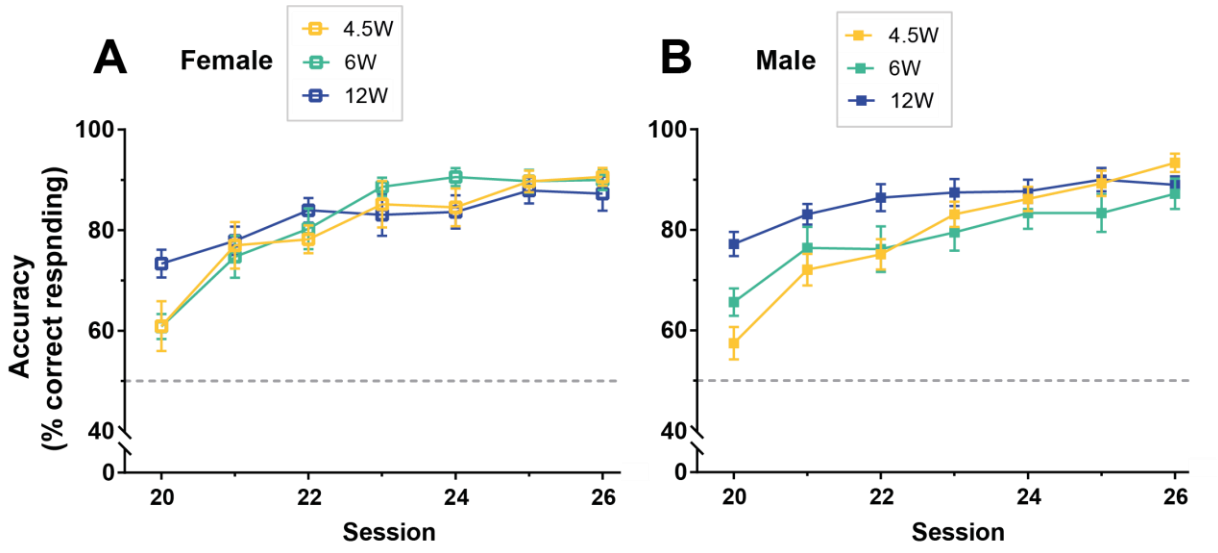

Supplemental Figure S5: sPAL memory retention following a rest period.

(A) Female and (B) male mice showed age-dependent differences on the first session response accuracy following a 2-week rest period (session 20) (see also Figure 5) but returned to 85-90% response accuracy following addition sessions. Data are mean ± SEM (4.5W: n=13 male, n=11 female; 6W: n=13 male, n=12 female; 12W: n=13 male, n=12 female). Gray dotted line indicates performance at chance (50% accuracy).

Supplemental Table S1. Sex-dependent effects on the number of sessions to complete touchscreen pretraining stages.

Number of sessions to criterion/ complete pretraining stage	3.5W ♂	3.5W ♀	5W ♂	5W ♀	11 ♂	11W ♀
**Stage 1:** Habituation	1.0 ± 0.0	1.0 ± 0.0	1.0 ± 0.0	1.0 ± 0.0	1.1 ± 0.1	1.0 ± 0.0
**Stage 2:** Initial Touch	1.0 ± 0.0	1.0 ± 0.0	1.0 ± 0.0	1.0 ± 0.0	1.0 ± 0.0	1.0 ± 0.0
**Stage 3:** Must Initiate	**1.1 ± 0.1**	**2.5 ± 0.5**	**1.1 ± 0.1**	**1.8 ± 0.5**	**1.0 ± 0.0**	**1.4 ± 0.4**
**Stage 4:** Punish Incorrect	3.7 ± 0.3	3.3 ± 0.5	2.3 ± 0.3	2.8 ± 0.4	3.5 ± 0.4	3.6 ± 0.4

Male (♂) and female (♀) mice commenced touchscreen pretraining at 3.5W, 5W and 11W of age and progressed through Stages 1 and 2 within comparable numbers of sessions to criterion. When animals reached Stage 3 that required mice to self-initiate the commencement of trials and make an instrumental operant nose-poke response to stimuli in order to receive rewards (Must Initiate), female mice at all 3 ages but particularly early-adolescence (3.5W) required significantly more sessions of training on this phase compared to age-matched male mice. Data are mean number of sessions ± SEM (4.5W: n=13 male, n=11 female; 6W: n=13 male, n=12 female; 12W: n=13 male, n=12 female).

Supplemental Table S2: Variables included in regression models

Dependent variable	Statistical model	Independent variables	Effect size	Lower CI.	Upper CI.	P value	Corresponding Figure
Trial outcome in sPAL (correct or incorrect)	Mixed-effect generalized linear model (logit link)	6W	0.83	0.67	1.02	0.077	Figure 3D, E
		4.5W	0.75	0.61	0.90	0.003	
		Sex (male)	0.96	0.81	1.12	0.587	
		Session	1.15	1.14	1.16	< 0.001	
		Trial within session	1.00	1.00	1.00	0.097	
		Correct location (right)	0.91	0.83	1.00	0.062	
		6W x Sex (male)	1.20	0.79	1.80	0.393	
		4.5W x Sex (male)	1.31	0.89	1.92	0.171	
		6W x Session	0.97	0.95	0.99	0.007	
		4.5W x Session	0.97	0.95	0.99	0.003	
		Session (12W)	1.17	1.16	1.19	< 0.001	
		Session (6W)	1.14	1.12	1.16	< 0.001	
		Session (4.5W)	1.14	1.12	1.15	< 0.001	
Trial outcome in sPAL (correct or incorrect) session≤10	Mixed-effect generalized linear model (logit link)	6W	1.04	0.86	1.27	0.675	Figure S4
		4.5W	1.17	0.99	1.40	0.072	
		Sex (male)	0.97	0.82	1.14	0.695	
		Session	1.18	1.13	1.23	< 0.001	
		Trial within session	0.9978	0.9962	0.9995	0.012	
		Correct location (right)	0.91	0.82	1.02	0.096	
		6W x Session	0.97	0.92	1.03	0.361	
		4.5W x Session	0.92	0.87	0.97	0.002	
Trial outcome in sPAL (correct or incorrect) 11≤session≤19	Mixed-effect generalized linear model (logit link)	6W	0.72	0.45	1.15	0.166	Figure S4
		4.5W	0.64	0.42	0.97	0.034	
		Sex (male)	0.95	0.76	1.18	0.625	
		Session	1.11	1.04	1.18	0.001	
		Trial within session	1.006	1.002	1.011	0.004	
		Correct location (right)	0.90	0.75	1.08	0.256	
		6W x Session	0.99	0.92	1.08	0.897	
		4.5W x Session	1.01	0.94	1.10	0.739	
Memory retention in sPAL (correct or incorrect)	Mixed-effect generalized linear model (logit link)	6W	0.63	0.50	0.81	< 0.001	Figure 4C
		4.5W	0.59	0.47	0.74	< 0.001	
		Sex	0.96	0.79	1.17	0.714	
		Retention	0.28	0.23	0.33	< 0.001	
		Trial within session	1.01	1.00	1.01	0.028	
		Correct location (right)	0.82	0.67	1.00	0.046	
		6W x Retention	0.75	0.51	1.11	0.156	
		4.5W x Retention	0.62	0.41	0.94	0.026	
		Retention (12W)	0.35	0.27	0.47	< 0.001	
		Retention (6W)	0.27	0.20	0.36	< 0.001	
		Retention (4.5W)	0.23	0.17	0.31	< 0.001	
Initiation latency in sPAL	Quantile regression (median)	6W	0.99	0.48	1.51	< 0.001	Figure 5G
		4.5W	1.74	1.14	2.33	< 0.001	
		Sex (male)	-0.36	-0.42	-0.31	0.711	
		Session	0.09	-0.37	0.54	< 0.001	
		Trial within session	0.00	-0.01	0.00	0.298	

		Correct location (right)	-0.03	-0.13	0.07	0.571	
		Correction trial	-0.69	-0.93	-0.45	< 0.001	
		6W x Sex (male)	0.10	-0.91	1.10	0.849	
		4.5W x Sex (male)	-1.04	-2.16	0.08	0.068	
Stimulus-approach latency in sPAL	Quantile regression (median)	6W	0.07	0.01	0.12	0.022	Figure 5H
		4.5W	0.07	0.01	0.14	0.027	
		Sex (male)	0.05	0.00	0.10	0.069	
		Session	-0.03	-0.03	-0.03	< 0.001	
		Trial within session	0.00	0.00	0.00	< 0.001	
		Correct location (right)	0.00	-0.01	0.01	0.535	
		Correction trial	0.03	0.01	0.04	< 0.001	
		6W x Sex (male)	-0.03	-0.14	0.09	0.641	
		4.5W x Sex (male)	-0.13	-0.25	-0.01	0.040	
Reward collection latency in sPAL	Quantile regression (median))	6W	-0.01	-0.06	0.05	0.804	Figure 5F
		4.5W	0.11	0.05	0.17	0.000	
		Sex (male)	0.03	-0.02	0.08	0.207	
		Session	-0.02	-0.02	-0.02	0.000	
		Trial within session	0.00	0.00	0.00	0.616	
		Correct location (right)	-0.01	-0.03	0.01	0.402	
		Correction trial	-0.02	-0.04	-0.01	0.002	
		6W x Sex (male)	-0.05	-0.15	0.05	0.357	
		4.5W x Sex (male)	-0.10	-0.22	0.01	0.080	
Slope of latency vs. accuracy correlation	Linear regression	Initiation latency	-1.57	-2.84	-0.29	0.017	Figure 5J – L
		Stimulus approach latency	-28.69	-45.73	-11.66	0.001	
		Reward collection latency	-24.15	-44.14	-4.17	0.019	
